# Experimental study on the deformation localisation and acoustic emission characteristics of coal in Brazilian splitting tests

**DOI:** 10.1038/s41598-022-10332-7

**Published:** 2022-04-15

**Authors:** Wei-yao Guo, Wei Zhang, Cheng-guo Zhang, Yang Chen

**Affiliations:** 1 College of Energy and Mining Engineering, Shandong University of Science and Technology, Qingdao, 266590 China; 2 State Key Laboratory of Mining Disaster Prevention and Control Co-Founded By Shandong Province and the Ministry of Science and Technology, Shandong University of Science and Technology, Qingdao, 266590 China; 3 School of Minerals and Energy Resources Engineering, UNSW Sydney, Sydney, Australia; 4 Shandong Energy Group Co., Ltd., Jinan, 250014 China

**Keywords:** Geology, Fossil fuels

## Abstract

This paper presents an experimental study to assess the behaviour of coal samples under tensile loadings to better understand the failure mechanisms and the interactions with the coal characteristics. A set of Brazilian splitting tests were carried out using disk specimens obtained from Tashan Coal Mine in China. The digital speckle correlation method and acoustic emission (AE) were used to capture the deformation localisation and AE characteristics of each specimen during the loading process. The precursor characteristics of AE and the failure mechanism are discussed. It was found that the entire loading process mainly consists of compaction, elastic and post-peak dropping stage without an obvious yielding stage. Two kinds of deformation localisation were observed: central symmetry and axis symmetry. The corresponding AE evolution patterns have different phases, including gradual rise, step rise, transient rise and steady rise. During the subcritical failure stage, AE counts demonstrate a “rapidly increasing + flatten” intermittent feature. The results provide a reference for a better understanding of the damage process of the brittle coal material and its application in ground control design.

## Introduction

Rock and coal are distinguished from other engineering materials by their low tensile strength. Since the tensile strength of coal is much lower than its compressive strength, many coal structural failures are triggered by the tensile strength acting on part of the structure or the entire structure^1,2^. The tensile strength of coal is one of the commonly used parameters in ground support design and other mining geotechnical analyses. The ground conditions of mine roadways and the occurrence of dynamic failures are closely related to the inherent failure mechanisms of coal material^3–6^. Therefore, an improved understanding of the failure mechanisms under tension for coal is essential to determine the overall failure modes of coal mass and evaluate the long-term stability of rock mass for the design and management process in geotechnical engineering.

The commonly used methods for measuring the tensile strength of rock and coal samples include the direct tensile test and splitting test. Because there are some difficulties in conducting the direct tensile test, such as clamping, centring and bending moment eliminating, the Brazilian splitting test is widely used instead. The Brazilian test was first introduced to measure the tensile strength of cylindrical concrete samples^7^. According to the Griffith theory, crack initiation occurs in the centre of the disk to ensure that maximum transverse stress^8,9^. There have also been studies that observed that fractures start close to the loading zone^10–12^. A sudden failure of the specimen usually occurs at the end of the tests^13^. In recent years there has been an increasing number of studies to investigate the role of a range of contributing factors on the specimen tensile strength, including the shape and size effects, anisotropy, heterogeneity, boundary conditions etc.^14,15^. Apart from rock material, Brazilian tests have also been widely used for the testing of concrete specimens under different loading conditions^16–19^. As for weak rock, e.g. coal, many studies have been conducted on the factors affecting the tensile failure characteristics and deformation localisation of coal using the Brazilian splitting method^20–23^. When the coal disk specimens with different layer orientations are loaded, the tensile strength for a parallel layer is larger than that for the vertical layer, but the data dispersion is smaller^24^. It is also found that the coal disk specimen not only breaks along the axial loading direction, but also shears along the cleating path. The load–control loading can easily cause brittle failure, and the displacement–control loading might lead to step dropping after peak stress^25,26^.

Acoustic emission (AE) measurements can reveal the failure mechanism of coal under tensile conditions. The process of internal crack initiation, propagation and coalescence can be deduced by analysing the time, space and fracture strength of AE signals in the Brazilian splitting test. The AE characteristics during the Brazilian splitting test have been extensively investigated in the literature. The Queen’s University Seismology Laboratory conducted experimental work combining the ultrasonic tomographic imaging and acoustic emission techniques to examine stress changes and microfracture activities during the Brazilian test of Lac du Bonnet granite^27,28^. Labuz et al.^29^ utilised 3D AE locations to analyse the failure of the high strength concrete samples. In addition, rock composition affects the cracking pattern and fracturing mechanisms that have a close relationship with AE signal feature parameters^30,31^. Furthermore, the signal frequency is one of the critical factors to study the stress changes and failure mechanisms of the specimen^32,33^. There have also been many recent studies using AE monitoring techniques to examine the failure processes of coal specimens. For example, Liang et al.^34^ studied the failure process of coal specimens of different types and sizes under tensile conditions using AE. Shkuratnik et al.^35^ examined the AE characteristics of coal specimens under triaxial loading. Chen et al.^36^ studied the attributes of time, space and fracture strength of AE events using the numerical modelling method. Li et al.^37^ verified the existence of the Kaiser effect in the Brazilian splitting test. Additionally, Jin et al.^38^ researched the influences of loading contact and platen size on the Brazilian splitting test. Li et al.^39^ analysed the relationship between tensile strength and thickness–diameter ratio based on a large number of tests. Su et al.^40^ conducted Brazilian splitting tests under impact conditions and discussed the effects of burst velocity and bedding angle on the energy density and damage variable of coal specimens. In addition, the digital speckle correlation method (DSCM) has a high resolution of strain measurement and can monitor the failure process of coal^41–43^.

This paper uses AE and DSCM methods to systematically study the splitting process of coal disk specimens obtained from a mine in Shanxi Province, China. The deformation localisation and AE activity of coal disk specimens are analysed, then the fracture mechanism and subcritical failure modes of the specimens are discussed.

## Brazilian splitting test setup

### Experiment specimen preparation

**Figure 1:**
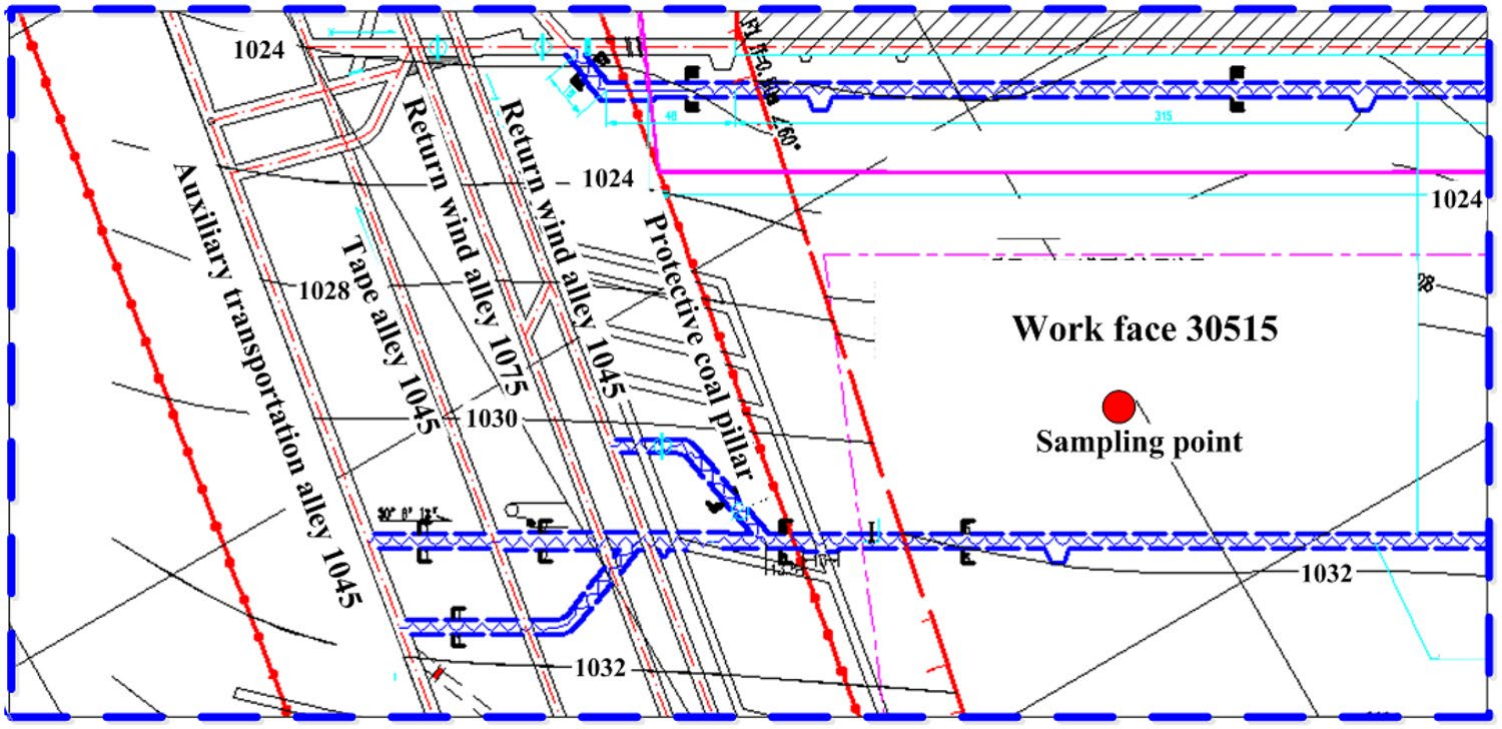
The location of the longwalls where the coal samples were taken.

The coal samples were obtained from the No. 3–5 coal seam in Tashan Coal Mine in Shanxi Province, China (Fig. 1). The thickness of the coal seam is 15.72–26.77 m with an average value of 17.93 m. The stratigraphic structure of the coal seam is complicated, consisting of 5–11 partings of kaolin or carbon mudstone. The immediate roof is kaolin mudstone with a thickness of 0.79–6.67 m, overlaying by the sandstone in the main roof. The immediate floor is sandy mudstone with a thickness of 0.60–8.84 m.

**Figure 2:**
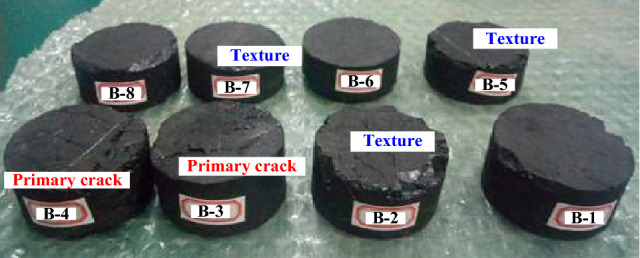
Overview of the test specimens.

The coal disk specimens were prepared according to the ISRM standards. The diameter and height of the specimens were 50 mm and 25 mm, respectively (Fig. 2). Table 1 lists the mechanical properties of the coal.

**Table 1:** Physical and mechanical properties of the coal.

Density (kg/m^3^)	Elastic modulus (GPa)	Uniaxial compressive strength (MPa)	Poisson ratio	Dynamic failure duration (ms)	Bursting energy index
1428	3.26	9.44	0.23	713.56	2.91

### Loading and monitoring system

**Figure 3:**
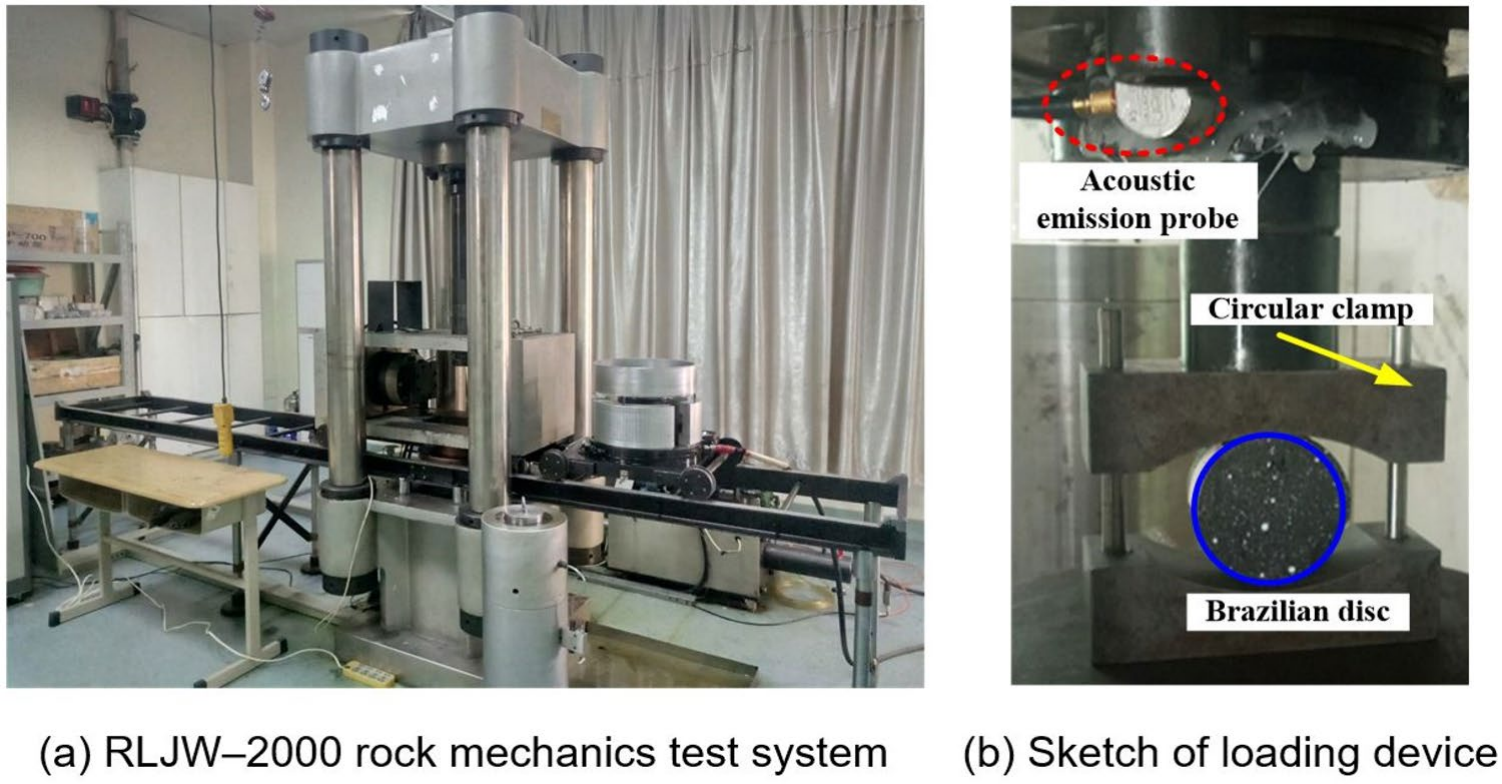
Testing system setup.

The tests were conducted using a RLJW-2000 servo-controlled rock pressure testing machine (Fig. 3), with a loading rate of 0.025 mm/min. The AMSY-6 AE monitoring system was used to record the AE signals. The sampling frequency was 5 MHz, with the threshold of the AE probe of 35 dB, and pre-amplifier gain of 60 dB. The high pass and low pass of the AE probe were set to be 1 kHz and 800 kHz, respectively.

**Figure 4:**
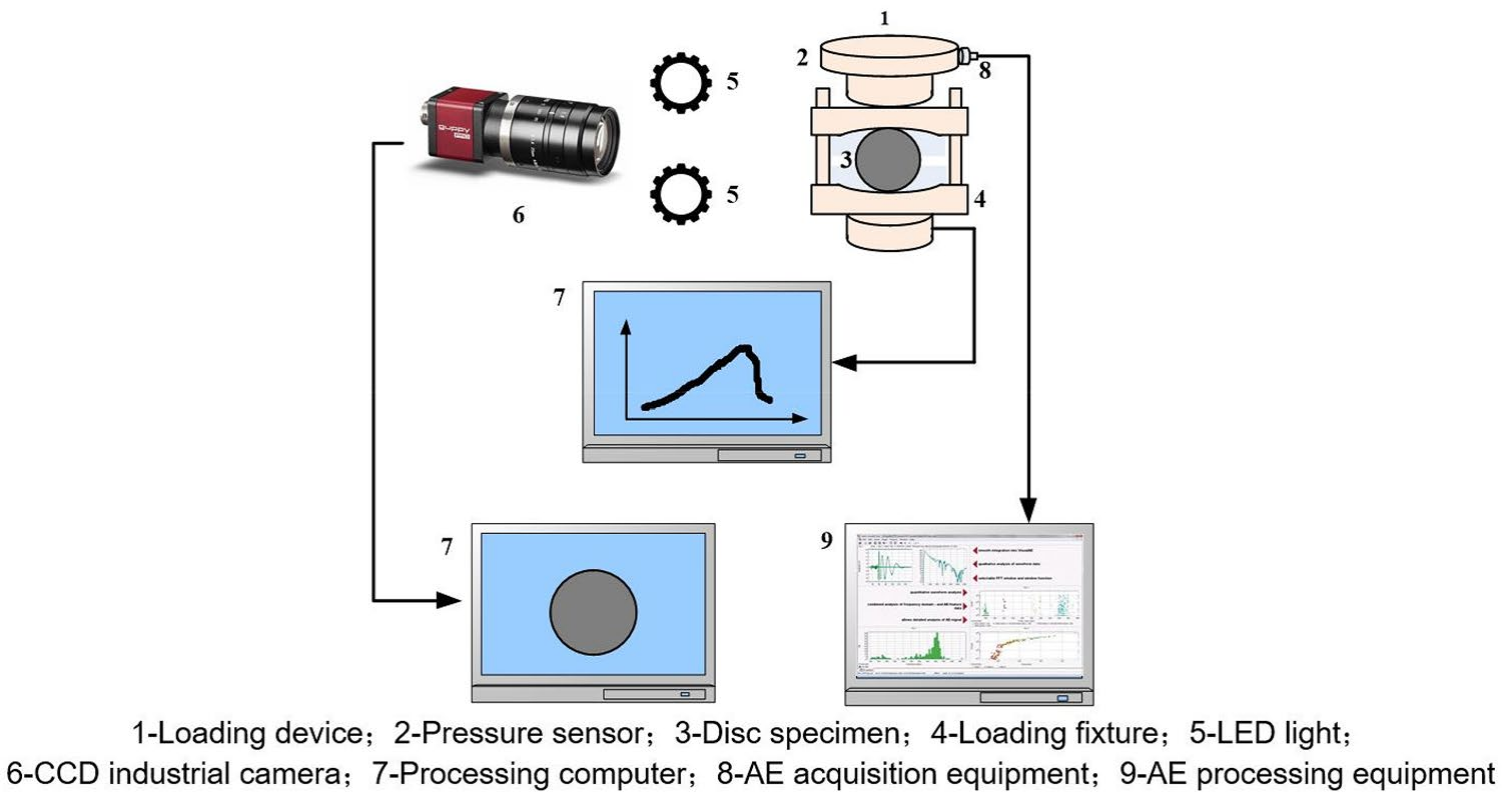
Digital speckle test system.

To obtain the horizontal displacement field of the specimen surface, a CCD industrial camera with an effective pixel of 2048 × 1536 was used. The digital speckle system is illustrated in Fig. 4. LED coal light sources were placed on both sides of the specimen. During the test, the optical axis of the CCD camera was perpendicular to the specimen surface. The specimen surface was sprayed with artificial speckles. The evolution process of the deformation field was obtained through post-processing of the collected images.

## Test results and analysis

### Deformation localisation

**Figure 5:**
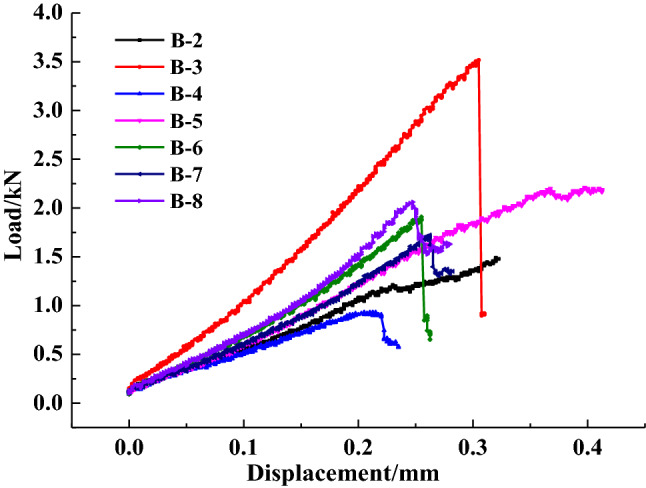
Load–displacement curves for the specimens.

The load–displacement curves of the seven specimens are presented in Fig. 5. Specimen B-1 was excluded as it failed when it was being set up in the test. The entire loading process mainly consists of an initial compression stage, elastic stage and post-peak stage without prominent yield characteristics, showing the typical brittleness failure features.

**Figure 6:**
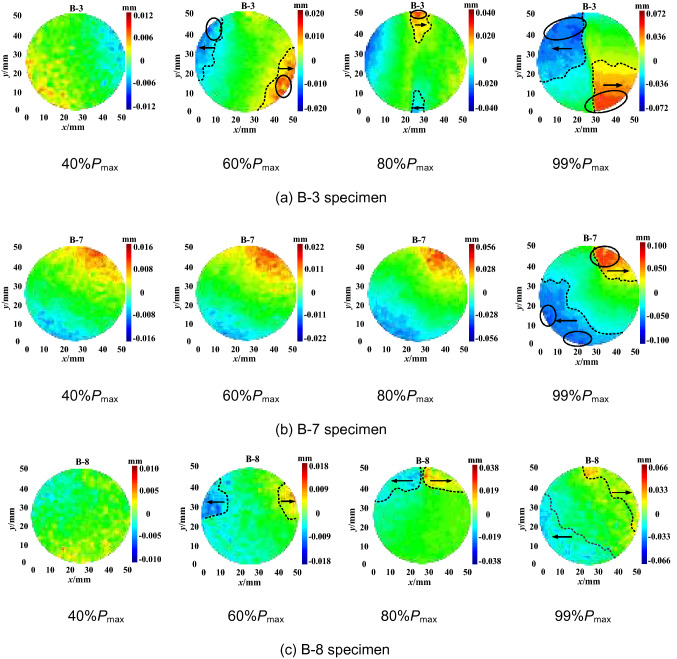
Horizontal displacement field of central symmetry.

**Figure 7:**
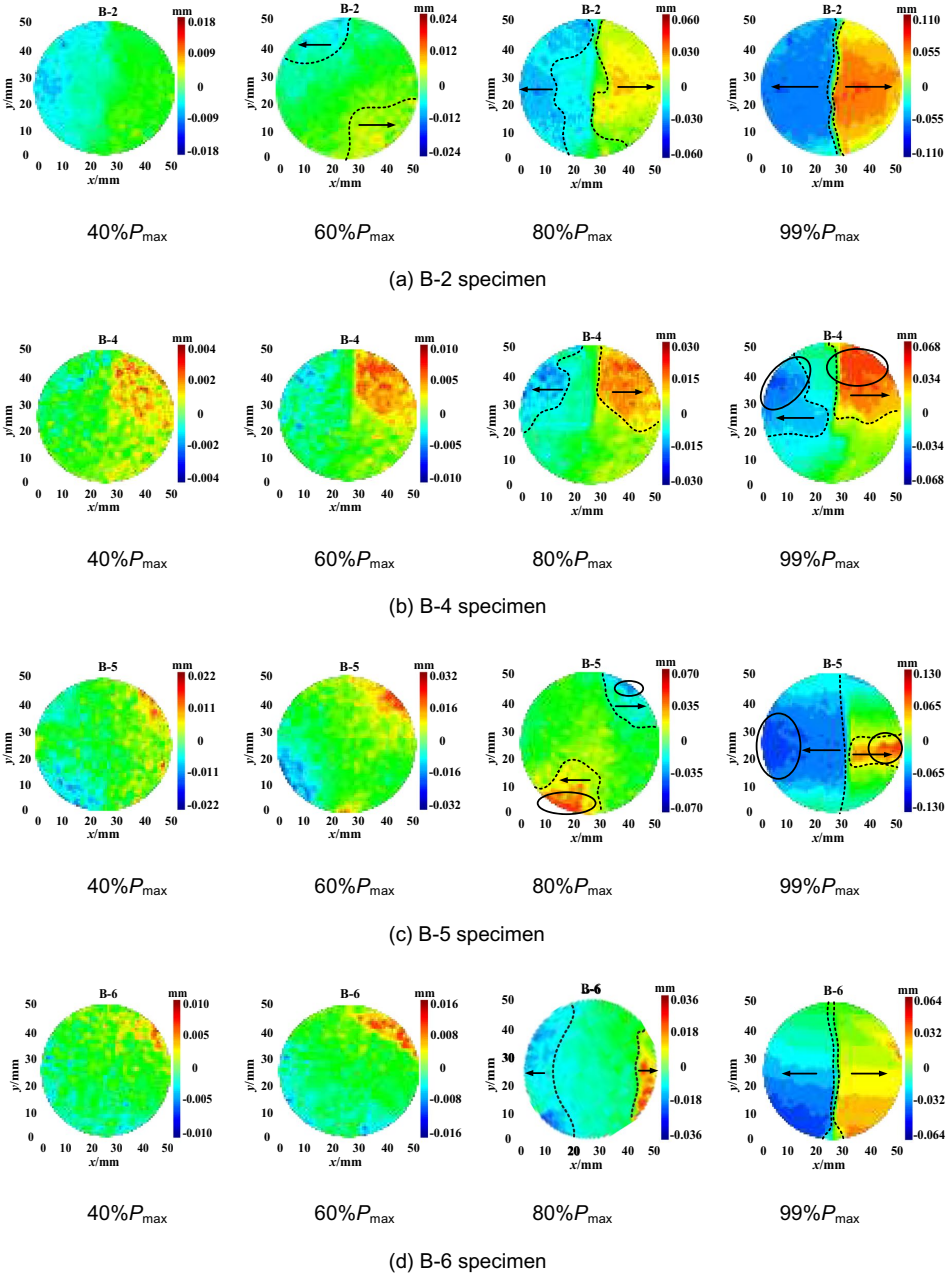
Horizontal displacement field of axis symmetry.

The horizontal displacement measurement of the specimens shows that there are two types of deformation localisation and distribution pattern, i.e. central symmetry and axis symmetry, as shown in Figs. 6 and 7, respectively. As shown in Fig. 6, when the load reaches 40% of the maximum tensile strength *P*_max_, the horizontal displacement increases gradually, and deformation localisation initiates. As the load continues increasing to 60% of the *P*_max_, the trend of central symmetry occurs. In particular, the deformation concentration was clearly observed at the two ends of the specimen. When the load increases to 80% of the *P*_max_, the maximum displacement increases significantly. For example, the maximum displacement of the B-7 specimen increases from 0.022 mm to 0.056 mm. The displacement reaches the maximum at the peak load. The extreme region of horizontal displacement mainly distributes at the two ends of the specimen. However, the distribution pattern varies for different groups. The B-3 specimen shows a fan-shape symmetry while the B-7 and B-8 specimens have irregular band symmetry. As shown in Fig. 7, the changing distribution pattern of the horizontal displacement of the specimens is similar to that of the central symmetry. Deformation localisation and rapid increase of displacement were also observed. The main difference compared to the central symmetry is that the horizontal displacement is distributed symmetrically on the two sides of the specimen axis. Moreover, macro cracks developed through the upper and lower ends of the specimen. This indicates that the crack propagation mode could affect the change of the horizontal displacement distributions, i.e. the development type of horizontal displacement reflects the crack variation characteristics.

Overall, the DSCM results demonstrate the horizontal displacement development pattern in the Brazilian splitting test as follows. No cracks was observed on the specimen surface during the early loading process (0–40% of the *P*_max_). As the load linearly increases in the elastic deformation stage, the region of the horizontal displacement field and the position of maximum displacement basically concentrates in close proximity to the specimen edge. As the load increases to 40–60% of the *P*_max_, deformation localisation occurs. The maximum displacement gradually extends to the two ends of the specimen parallel to the loading direction. The deformation localisation effects are relatively consistent at this stage. However, when the cracks propagate at 60–80% of the *P*_max_, the growth rate of deformation localisation accelerates. The steady crack propagation transforms into unstable propagation. The region of deformation localisation rapidly extends to the end position of the specimen. At 80–99% of the *P*_max_, the width of deformation localisation band increases, revealing the occurrence of macro crack extension. When the load reaches the maximum value, the macro cracks rapidly penetrate the specimen in a short period accompanied by significant stress drop.

### AE characteristics analysis

**Figure 8:**
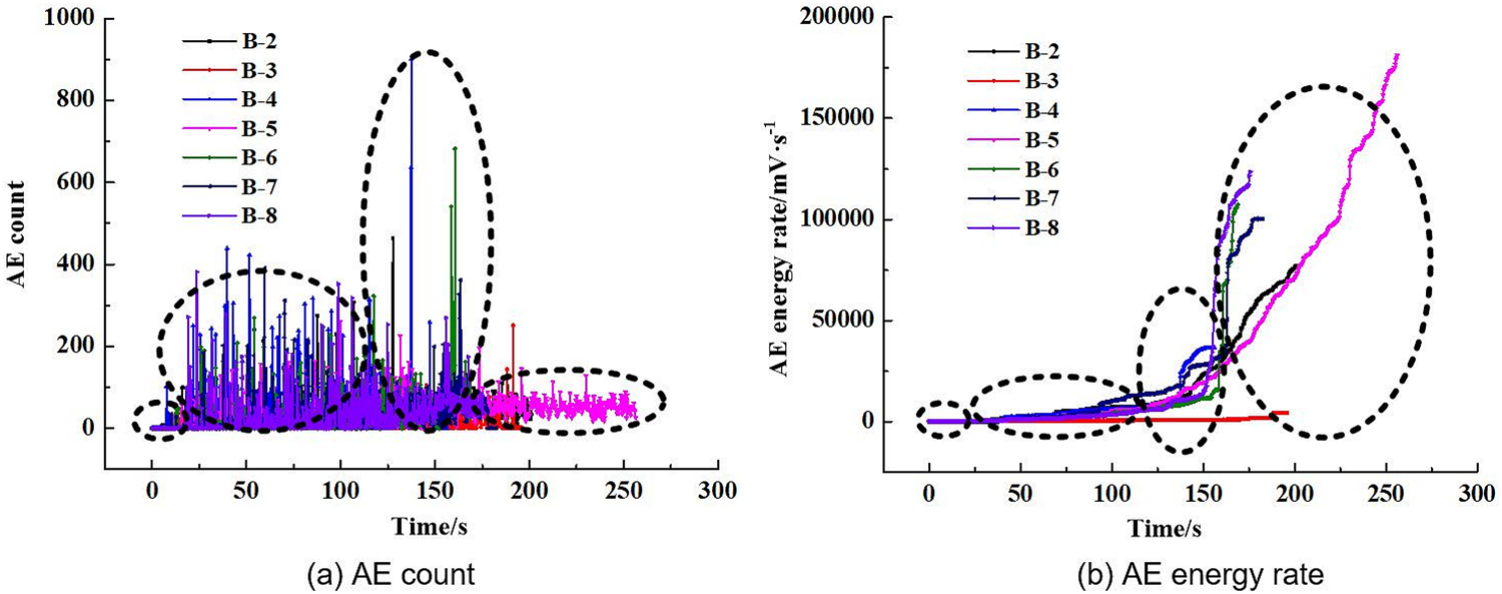
AE count, energy rate–time curve.

**Figure 9:**
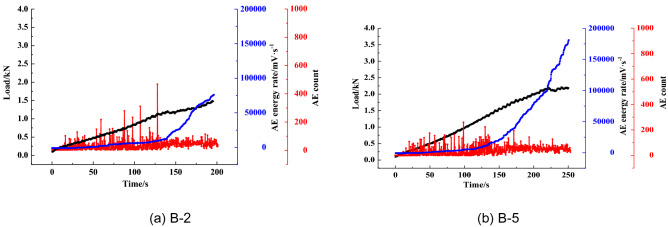
Gradual rise type.

**Figure 10:**
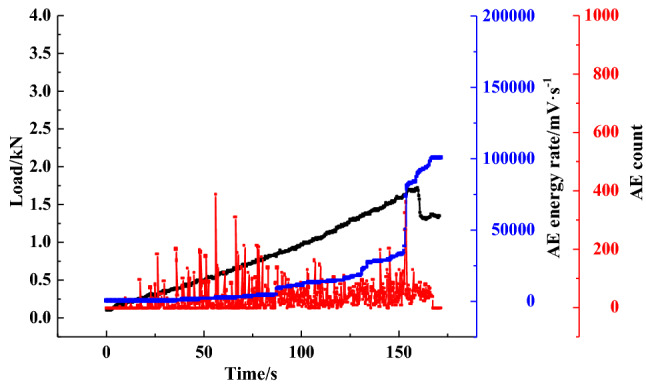
Step rise (B-7).

**Figure 11:**
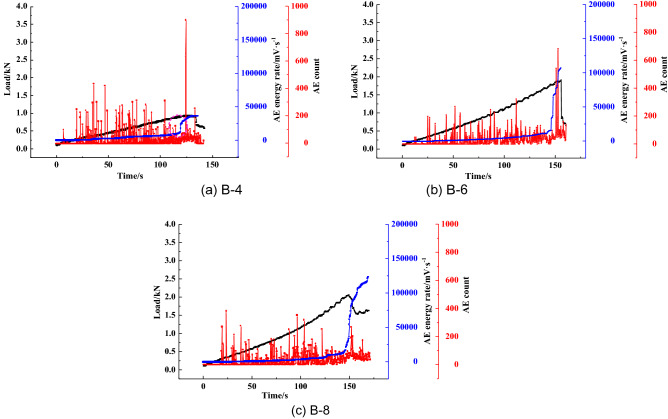
Transient rise type.

**Figure 12:**
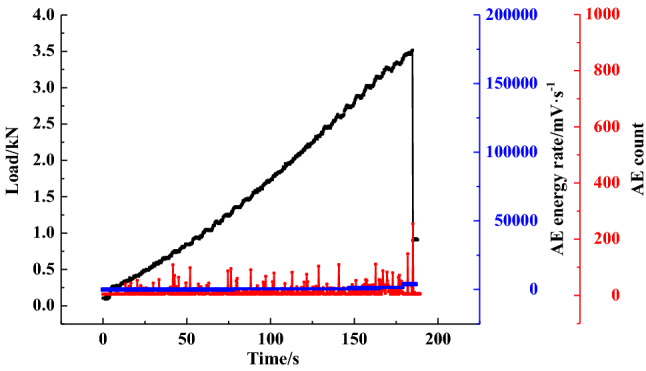
Steady rise (B-3).

Figure 8 illustrates the changes in the AE cumulative counts and energy rate for the coal specimens in the loading process. The graph shows that during the initial compaction stage, the AE counts and energy rate are at a low level. When the samples enter the elastic stage, the AE energy rate experiences a slight increase, and the AE counts reach a high level. At 85% of the *P*_max_, the AE counts and energy rate show significant growth due to the bilateral dislocation of cleats, which were also observed in the literature^41^. When the specimen fails, the AE energy rate sharply increases, reaching the peak value, followed by the stress drop and the rapid decrease of AE cumulative counts. AE counts then decrease back to the original level prior to the failure. In addition, it was also found that the AE evolution characteristics are different. The AE results show that the AE evolution types can be divided into a gradual rise, step rise, transient rise and steady rise, as illustrated in Figs. 9, 10, 11, 12.

As shown in Fig. 9(a), the AE energy rate varies uniformly first and then accelerates until the specimen fails. Meanwhile, AE counts mainly concentrate in the early and middle loading stages, and the maximum value is reached before the AE energy rate rapidly increases. The reason is that the coarse and fine particles are evenly distributed in the early loading stage. The internal structure is relatively stable at this phase. In the next loading stage, the inner structure fails due to the decrease of particle bonding strength. As the load reaches the maximum, the specimen does not lose its bearing capacity completely, and the internal fracture continues until the full penetration of macro cracks. This result causes the “ductility” phenomenon in the splitting process. The AE characteristics of the B-5 specimen are similar to B-2, as shown in Fig. 9(b). The only difference is that the peak load and AE energy rate of the B-5 specimen are larger than that of the B-2 specimen, as the internal structure of the two samples is similar, but the B-5 specimen appears to have more cohesive cleating.

Figure 10 presents the AE and loading relationship for specimen B-7. The AE energy rate–time curve experiences three rapidly increasing stages, indicating that three violent fractures occur inside the specimen. The first two steps are caused by the cementation failure between particles and the appearance of a large number of micro cracks. The third step appears in the adjacent failure due to the internal structure failure. Additionally, the AE energy rate still increases after the peak loading point, suggesting the effect of residual fracturing.

The AE counts of the B-4, B-6 and B-8 specimens are uniformly distributed, as shown in Fig. 11, indicating that the propagation and expansion of internal micro cracks gradually occur. The mineral particles are evenly broken before the specimen failure, causing certain damage and microfracture. The variation of the corresponding energy rate–time curve is also uniform without any stepping phenomenon. Subsequently, the activity of crack generation and propagation is more frequent. A large number of micro cracks are generated and connected. When the load reaches the peak value, the AE energy rate rapidly reaches the maximum, and the splitting failure occurs in a short time. As illustrated in the AE count–time curve of the B-8 specimen, there is a significant difference compared to B-4 and B-6 specimens. The number of AE counts of the B-8 specimen at the peak load is smaller than that of the B-4 and B-6 specimens. It indicates that the factors affecting the AE characteristics are not only the composition and cementation, but also the internal structure.

The AE characteristics of the B-3 specimen show a significant difference compared to other specimens, where the AE energy rate threshold of the data is the smallest. As shown in Fig. 12, AE activity of the B-3 specimen evenly occurs before the sample reaches the peak load. The AE energy rate and AE counts remain below 10^4^ mV·s^−1^ and 200, respectively. The uniform internal properties might cause this phenomenon.

The AE count characteristics of the four tested types are listed in Table 2. The AE activity of the transient rise is the strongest, followed by the step rise; the gradual rise and the steady rise are on the weak side. The variation magnitude of AE counts is relatively large in the peak load for the transient rise type, indicating intense energy release during the splitting failure.

**Table 2:** Statistics of AE counts.

Specimen number	AE evolution type	AE counts per second	Maximum AE counts	Percentage of the time (the number of AE counts is larger than 120) (%)
B-4	Transient rise	230	910	33
B-6		175	690	24
B-8		180	410	19
B-7	Step rise	160	405	22
B-2	Gradual rise	145	480	15
B-5		130	230	12
B-3	Steady rise	50	210	5

### AE response during the crack propagation process

**Figure 13:**
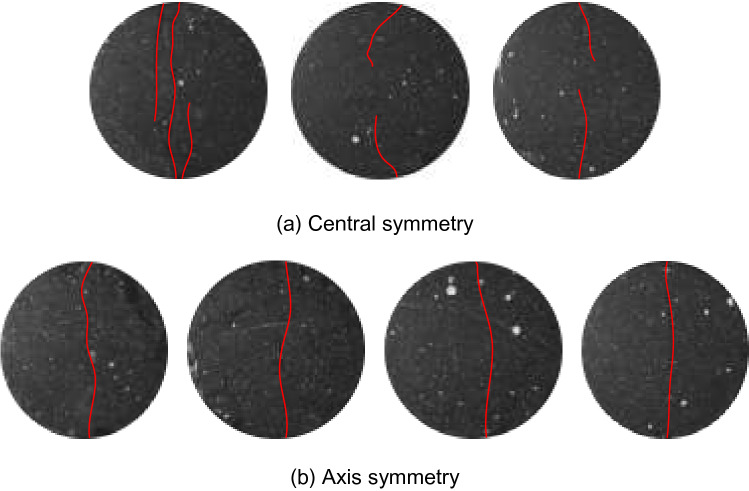
Sketch of crack propagation.

**Figure 14:**
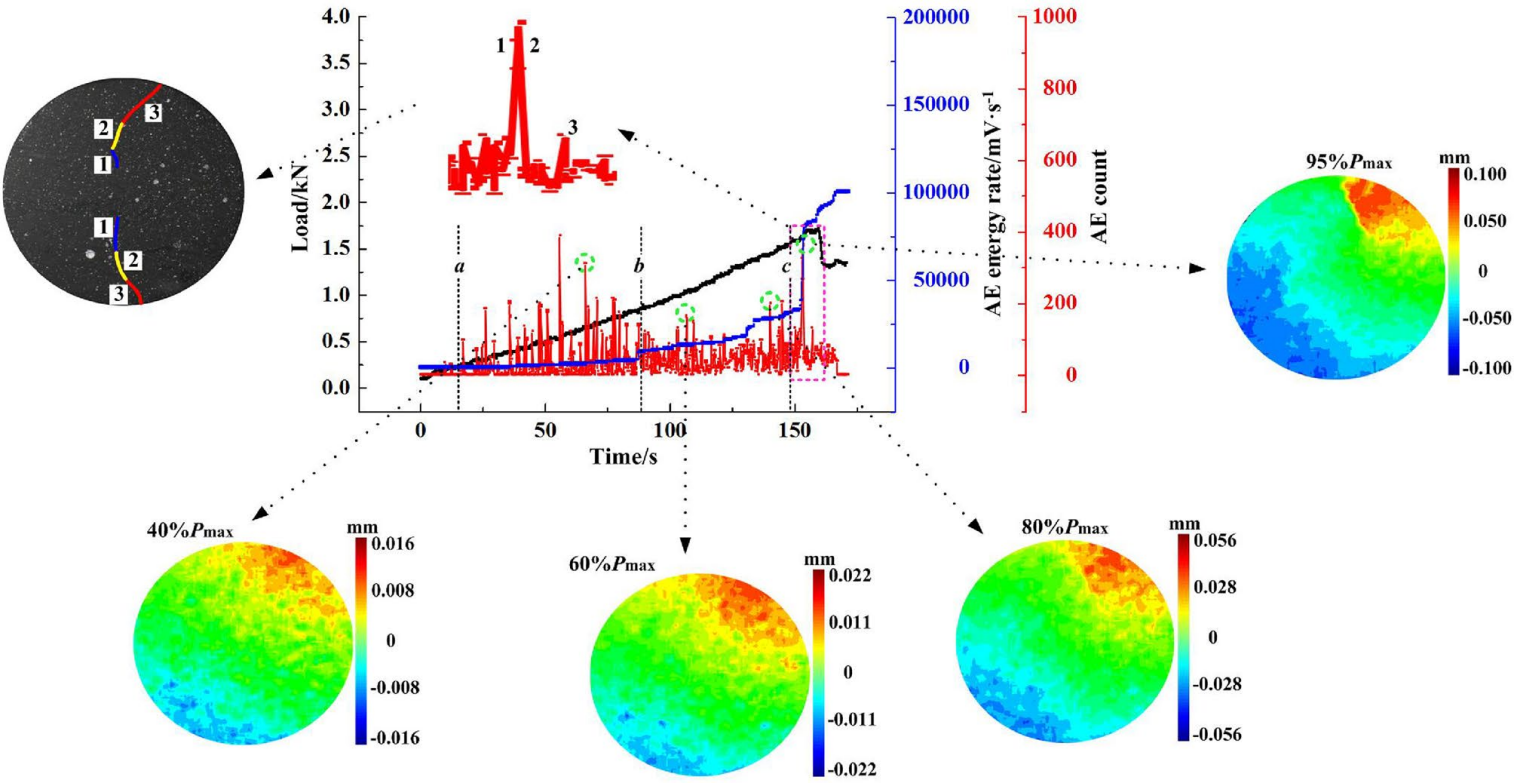
Central symmetry (B-7).

**Figure 15:**
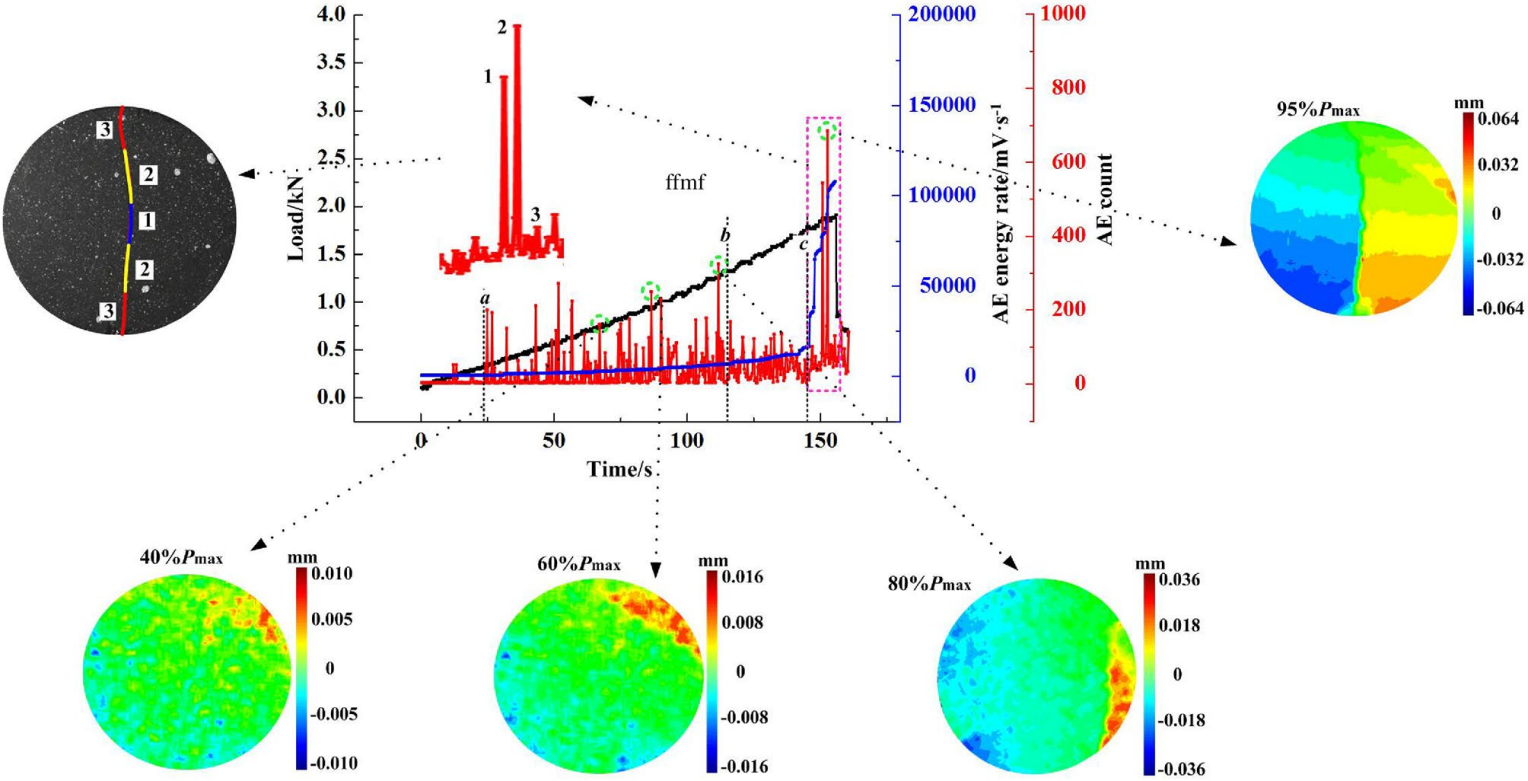
Axis symmetry (B-6).

The AE characteristics were further analysed, corresponding with the crack propagation process. As shown in Fig. 13, there are two types of crack propagation patterns: central symmetry and axis symmetry. The corresponding crack propagation process and AE response are illustrated in Figs. 14 and 15, respectively. AE counts show an overall increasing trend as the load increases. Additionally, AE counts show obvious “rapidly increasing + flatten” intermittent features in the subcritical instability stage.

Figure 14 illustrates the central symmetry failure mode of the B-7 coal specimen. At the initial loading stage before point a, the AE activity is not active. No macro cracks developed on the specimen surface. When the loading increases to point *a*, the horizontal displacement gradually increases to 0.012 mm. The horizontal displacement field at 40% of the *P*_max_ indicates that the specimen surface has no distinct fracture characteristics. The corresponding AE energy rate remains at a low level of 1.2 × 10^4^ mV·s^−1^, indicating that the specimen is still in the energy accumulation stage prior to the failure. The average number of AE events is 165, staying at a high level. The phenomenon might indicate that the energy source is widely and randomly distributed inside the specimen. As loading increases to point b, AE activity tends to be flat, and the number of AE events decreases to 105. The energy source begins to converge, which is consistent with the phenomenon of horizontal displacement concentration. At the same time, the AE energy rate shows a step rise trend, but at a low level of 1.7 × 10^4^ mV·s^−1^. This phenomenon suggests that the energy stored in the imminent fracture area further increases. At the loading point c, the specimen enters into the fracture stage accompanied by the gathering of micro cracks. The horizontal displacement increases to 0.045 mm. The displacement concentration is intensified. The AE energy rate quickly increases from 1.2 × 10^4^ mV·s^−1^ to 7.7 × 10^4^ mV·s^−1^. AE activity is pronounced when the peak load is reached. Macro crack 1 forms in the axial region, and its corresponding AE events greatly increase. The internal cracks significantly propagate along this direction. However, macro crack 2 extends offset the loading direction. The relevant AE events also increase, which means micro cracks extending in this direction appear. Macro crack 3 follows the crack 2 direction until the main macro crack forms, but the number of AE events is much lower than that of crack 1 or 2. Thus, it can be concluded that the formation of macro crack 3 is not induced by the coalescence of micro cracks.

Figure 15 illustrates the axial symmetry failure mode of the B-6 specimen. Similar to the central symmetry, the axial symmetry also experiences the compaction stage (before point a), fracture source random distribution stage (between point a and point b), crack initiation stage (between point b and point c) and macro failure stage (after point c). The number of AE events corresponding to macro cracks 1 and 2 is at a high level of 650, but the number of events corresponding to macro crack 3 is only 140. Figures 14 and 15 show that macro crack 3 mainly extends to the upper and lower ends of the specimen with a certain inclining angle to the axis. It reveals that the accumulated energy substantially releases after the completion of macro crack 2 propagation. Therefore, when the macro crack 3 propagates, the energy is not sufficient to concentrate on the micro cracks in close proximity to the main crack. Macro crack 3 can only extend along the propagation direction of macro crack 2.

## Discussion

The short period prior to the specimen failure is defined as the subcritical failure stage in this paper. AE activity shows that multiple fluctuation phenomena occur in the subcritical failure stage, especially the “rapidly increasing + flatten” intermittent feature. Whether in laboratory testing or in field monitoring, the subcritical failure is essential for warning of a rock and coal mass instability disaster. Thus, this section focuses on the experimental analysis in the subcritical failure stage.

The occurrence mechanism of AE is mainly the micro-fracturing and plastic yielding (i.e., particle dislocation and fracture) before the macro-cracking. When the load continues, the stress field at the micro crack tip continues to increase, and the accumulated elastic strain energy also increases. When the released energy *G* from the crack propagation is sufficient to overcome the propagation resistance *R*, i.e. *G* ≥ *R*, the specimen enters the subcritical failure stage. As the strain continues increasing, the stress exhibits nonlinear variation deviating from the original linear trend. At this time, the AE signal depends not only on the variations of stress and stress rate, but also on the crack propagation rate ^10^. It is known from fracture mechanics that there is *G* ≥ *R* during the crack propagation process. However, when the crack length *c* does not reach the critical value, the increasing rate of *R* with *c* is more significant than that of *G* (i.e., *G*/*c* < *R*/*c*). Therefore, the magnitude of *G* and *R* becomes similar as *c* increases during the crack propagation process, but the crack propagation rate *c* decreases. When the c propagates until *G* < *R*, the crack propagation stops, accompanied by quiet AE activity. As the stress further increases, the energy inducing the micro-crack propagation reaches the critical threshold again, the instantaneous released energy causes the sudden increase of the strain, and the response of AE counts rapidly increases. When *G* is larger than *R*, the crack propagates again. If the stress and crack length still do not reach the critical values, the crack might stop. This cycle continues until the coal sample totally yields. Therefore, the crack propagation process has several intermittent features in the subcritical failure stage. The corresponding AE signals show multiple precursor phenomena of “rapidly increasing + flatten”. This feature is strengthened, especially when the main rupture approaches.

Splitting failure was observed within the specimen at the peak load. The crack propagation rate *G*/*c* reaches and even exceeds the resistance growth rate *R*/*c*. Meanwhile, the crack propagation speed sharply increases and enters unstable expansion. Macro rupture occurs, indicating a large amount of elastic strain energy instantaneously releases. However, the AE counts do not reach the maximum. The phenomenon suggests that when the macro cracks and micro cracks coalesce, there is not enough time for friction and slippage to occur internally. Thus, the macro cracks can only form along the existing cracks in the loading direction until specimen failure.

Ideally, the failure modes of these coal specimens should have vertically penetrating-type cracks. However, the central symmetry specimens have crescent-shaped and curve-shaped cracks. This might be caused by the existing angle between the bedding plane and the loading direction. The specimen failure is the result of a combination of tensile and shear stresses. Cai et al. ^44^proposed that the mixed failure mode and crack location should be fully considered for accurately conducting the Brazilian splitting test. Meanwhile, the effects of the deviation of specimen position and the end friction, causing no appearance of the penetrating-type crack, also need to be considered. In addition, the failure pattern and its associated mechanism could be further studied using a more complicated 3D AE monitoring.

## Conclusion

This paper assessed the mechanical response of coal under tensile loading conditions using Brazilian splitting tests on various types of coal disk specimens. The DSCM and AE techniques were used to assess the deformation localisation and AE characteristics. The main conclusions of this study are summarised as follows:

1. The entire loading process of the coal disk specimen consists of the compaction stage, elastic stage and post-peak dropping stage without an obvious yielding stage, exhibiting a brittle failure feature. The specimens all experience splitting failure, which is in good agreement with the horizontal displacement field observed in the samples.
2. There are two kinds of deformation localisation patterns found in this study: central symmetry and axis symmetry. The load mainly affects the spatial distribution characteristics of crack propagation.
3. The AE characteristics have gone through different stages as the loading increases. The AE evolution types include gradual rise, step rise, transient rise and steady rise. Especially at the subcritical failure stage, AE counts show a “rapidly increasing + flatten” intermittent feature.

The findings of this paper improved the understanding of the tensile failure mechanism of coal material. The results regarding the deformation localization pattern provide further insights into the crack initiation and propagation position of disc specimens, particular for weak rock. Overall, it can contribute to a better understanding of the mechanical behaviour of various types of coal for a potential improved rock engineering design.
